# Heterocyclic Compounds as Hsp90 Inhibitors: A Perspective on Anticancer Applications

**DOI:** 10.3390/pharmaceutics14102220

**Published:** 2022-10-18

**Authors:** Mina Ardestani, Zahra Khorsandi, Fariba Keshavarzipour, Siavash Iravani, Hojjat Sadeghi-Aliabadi, Rajender S. Varma

**Affiliations:** 1 Pharmaceutical Sciences Research Center, School of Pharmacy and Pharmaceutical Sciences, Isfahan University of Medical Sciences, Isfahan 81746-73461, Iran; 2 School of Pharmacy and Pharmaceutical Sciences, Isfahan University of Medical Sciences, Isfahan 81746-73461, Iran; 3 Regional Centre of Advanced Technologies and Materials, Czech Advanced Technology and Research Institute, Palacký University in Olomouc, Šlechtitelů 27, 783 71 Olomouc, Czech Republic

**Keywords:** Hsp90 inhibitor, co-chaperone, heterocycle molecules, anticancer agents, heat shock proteins

## Abstract

Heat shock proteins (Hsps) have garnered special attention in cancer therapy as molecular chaperones with regulatory/mediatory effects on folding, maintenance/stability, maturation, and conformation of proteins as well as their effects on prevention of protein aggregation. Hsp90 ensures the stability of various client proteins needed for the growth of cells or the survival of tumor cells; therefore, they are overexpressed in tumor cells and play key roles in carcinogenesis. Accordingly, Hsp90 inhibitors are recognized as attractive therapeutic agents for investigations pertaining to tumor suppression. Natural Hsp90 inhibitors comprising geldanamycin (GM), reclaimed analogs of GM including 17-AAG and DMAG, and radicicol, a natural macrocyclic antifungal, are among the first potent Hsp90 inhibitors. Herein, recently synthesized heterocyclic compounds recognized as potent Hsp90 inhibitors are reviewed along with the anticancer effects of heterocyclic compounds, comprising purine, pyrazole, triazine, quinolines, coumarin, and isoxazoles molecules.

## 1. Introduction

Cancer refers to a variety of diseases caused by escalating uninhibited cell proliferation, which may spread beyond tissue range. Cancer is initiated with the deformation of a normal cell caused by several hereditary factors, lifestyle, immune system defects, environmental/occupational factors, possible toxicity from medications, including the aging process, causing damage or genetic mutations [1,2,3,4]. The mechanisms of control, proliferation, and differentiation of cells are disrupted [5,6,7,8,9], which leads to deviation of normal cells from their regular growth path [10,11,12]. Tumor cells may acquire autonomy in two ways, namely activation of a growth-promoting oncogene or inactivation of a growth-inhibiting gene [13,14,15]. Cancer is one of the major death reasons which ranked after cardiovascular diseases; therefore, anticancer explorations have attracted much attention, especially through the evaluation of the deactivation of various proteins, such as tubulin, aromatase, and heat shock proteins [16,17,18,19,20,21,22,23,24].

Heat shock proteins (Hsps) inhibitors are one of the most eminent active anticancer agents with appropriate effects on several strange signaling paths in tumors; notably, these inhibitors can help to surmount several notorious problems regarding resistance cancers. Hsps act as proteins for vital cellular activities, including protein accumulation, secretion, and regulation of gene expression through direct correlation with transcription factors; the cellular expression is increased due to various stressors. Hsps are classified by their molecular weight into the Hsp110, Hsp90, Hsp70, Hsp60, Hsp40, and Hsp27; most of them being generally characterized as ATP-dependent [25,26,27]. Additionally, Hsp90 can regulate the activity and stability of different client proteins with a wide range of sizes and functions as these client proteins have critical roles in proliferation, survival, protein misfolding, aggregation, and apoptosis. There are four various homologs of Hsp90: cytosolic Hsp90 (including Hsp90α, Hsp90β), TRAP1 (tumor necrosis factor receptor-associated protein 1) in mitochondrial, GRP94 (94 kDa glucose-regulated protein) in the endoplasmic reticulum (ER), and Hsp90C in chloroplasts [27].

The crystal formation of Hsp90 was first defined in 1996 as a homodimer with a three-part monomer. The N-terminal domain (~35-kDa) is fabricated from layers of a/b sandwich structures formed in the pocket, acting as a binding site for adenine nucleotides. The requirement of ATP in Hsp90 is associated with auto-phosphorylation with the N-terminal folding pattern; the superfamily of ATPase has shown similar activity to Hsp90 by type II topoisomerases and MutL (Mutator L) [28,29].

**Figure 1:**
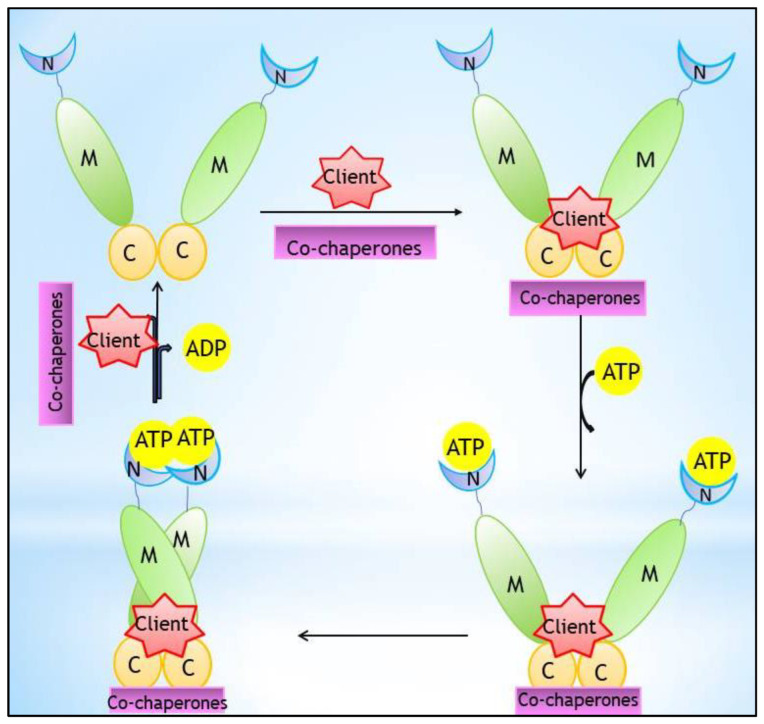
ATPase cycle in Hsp90.

A client protein binding site and nuclear localization signal, Hsp90 middle domain (~35 kDa), entails precise identification of client proteins and molecular regulator chaperones to activate the appropriate substrate, such as ATP hydrolysis [30]. The C-terminal domain (~12 kDa) is the site of dimerization close to the ATP binding site and a pentapeptide domain (Met-Glu-Glu-Val-Asp or MEEVD), as well as the binding site of co-chaperones of Hsp90 consisting of Sti15 and Hop [31]; the Hsp90 ATPase cycle is depicted in Figure 1. 

Hsp90 consists of a chaperoning subsidiary with the assistance of co-chaperones and ATP. Initially, the client protein attaches to the M domain with the co-chaperones source; afterward, the ATP binds to the N domain to dimer the Hsp90, leading to the production of “closed form” protein. The client protein is reformed, while the required energy is supplied from the bond division process, and ATP is hydrolyzed to ADP and unfastened phosphate. The release of client proteins and ADP from the complex occurs after covering the chaperoning function; ATP binds to the N terminal via a standard chaperoning cycle and then the client protein binds to Hsp90 by assisting co-chaperones [32,33,34].

Due to the unique effectiveness of Hsp90 inhibitors in cancer therapy, researchers have focused on them in recent decades [35,36,37,38,39,40,41,42,43,44,45,46,47]. Hsp90 is responsible for the conformational maturation of 500 client protein substrates embracing transcription factors, receptors, kinases, or oncoproteins, which might be overexpressed and/or mutated in most cancers [48]. Consequently, the inhibition of Hsp90 is contemplated an attractive cancer-treating strategy due to its impacts on oncoprotein and pathways, concurrently [48,49]. Herein, recent advances in potential inhibitory effects of lately synthesized heterocyclic compounds, such as purine, pyrazole, triazine, quinolines, coumarin, and isoxazoles against Hsp90, are deliberated, focusing on their anticancer and antitumor applications. 

## 2. Hsp90 Inhibitors

### 2.1. Natural Inhibitors

**Scheme 1:**
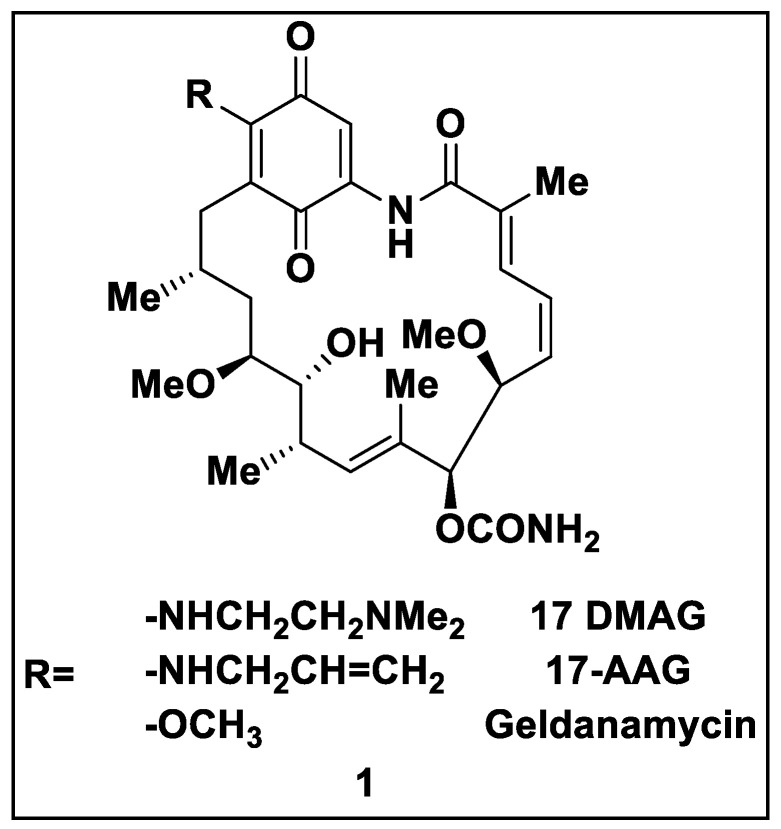
Chemical structure of geldanamycin and its analogs.

The first reported natural Hsp90 inhibitor has been a macrocyclic product called geldanamycin (**1**) (GM), extracted from the culture of *Streptomyces hygroscopicus* in 1970 which is known as an antibiotic compound (Scheme 1) [50]. Due to its unacceptable toxicological properties, the additional development of this compound was averted. However, the Hsp90 inhibitory activity of several geldanamycin analogs, namely, 17-dimethylaminoethylamino-17-demethoxygeldanamycin (17-DMAG), and 17-allylamino-17-demethoxygeldanamycin (17-AAG) are studied, but their clinical trials have been interrupted due to their poor solubility, complex formulation, and hepatotoxicity [51,52,53,54,55,56].

**Scheme 2:**
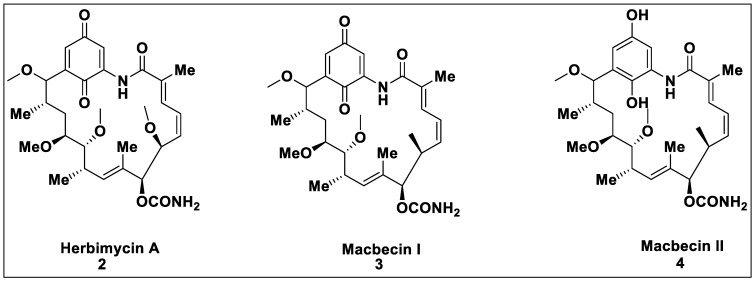
Chemical structures of herbimycin A, macbecin I, and macbecin II.

One of the developed GM analogs is herbimycin A in which the methoxy substitution is located at positions C-11 and C-15, and the methoxy group is not present at position C-17; GM and herbimycin A (**2**) can induce Hsp70 expression in fibroblasts [57]. Macbecin I (**3**) as a GM analog has been developed with a similar structure to herbimycin A, except that the methoxy group at C-6 is replaced by a methyl group. Macbecin II (**4**) is a hydroquinone analog of macbecin I with less separation tendency due to its inferior structural instability [58,59]. The chemical structures of Herbimycin A and macbecin (I and II) are depicted in Scheme 2.

**Scheme 3:**
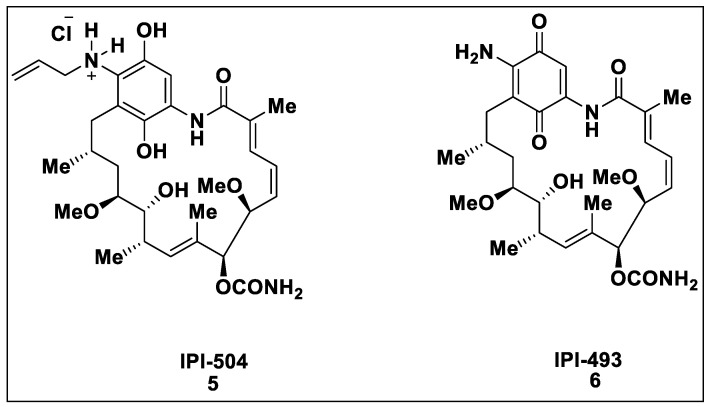
Chemical structures of IPI-504 and IPI-493.

IPI-504 (retaspimycin hydrochloride) (**5**) and IPI-493 (17-AG) (**6**) (Scheme 3) are more soluble analogs of GM and both of them inhibit Hsp90 activity through the degradation of client proteins. Several clinical trials (phase I and II) have been disclosed on different types of cancers including multiple myeloma, metastatic gastrointestinal stromal tumor (GIST), refractory non-small cell lung cancer (NSCLC), and chronic myelogenous leukemia (CML) [60]. IPI-493, equivalent to 17-AAG, obstructs the growth of SKBr3 breast cancerous cells, which may be derived from a similar major metabolite. IPI-493 exhibited highly effective results in GIST xenografts carrying heterogeneous KIT mutations in a preclinical investigation; these findings led to the start of its phase I clinical trial studies [61,62,63].

Benzoquinone ansamycins are also known as Hsp90 inhibitors that act as anti-malarial, antiviral, and anti-surra agents, and their efficacy in the treatment of cardiac arrest, stroke, and Alzheimer’s has been explored [64]. One of the natural Hsp90 inhibitors is Radicicol (RD) (**7**), which has been isolated from Monosporium bonorden, it can link to the N-terminal ATP binding site. RD is a potential cell growth inhibitor, but its unstable metabolite in the body leads to inactivity; therefore, elaborative studies have been focused on the modification of its structures to obtain analogs with better stability [65].

**Scheme 4:**
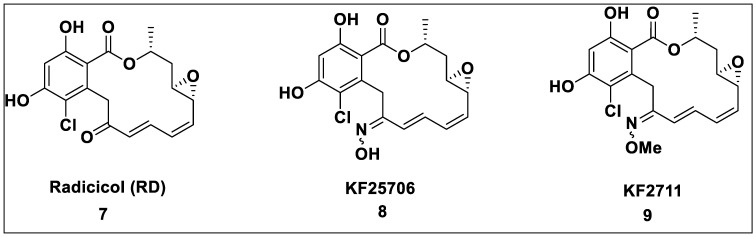
Chemical structures of Radicicol, KF25706, and KF2711.

KF25706 (**8**) and KF2711(**9**) have been introduced as more soluble analogs of RD with significant inhibition of the Hsp90 activity. As depicted in their chemical structure (Scheme 4), the carbonyl group in RD has been replaced with the oxime group, from the point of view of chemistry; this replacement can reduce its Michael acceptor electrophilicity thus improving the stability. However, their anticancer investigations have been limited to in vivo and animal model studies [65,66].

### 2.2. Synthetic Hsp90 Inhibitors

#### 2.2.1. Purine-Based Structures

Naturally-derived Hsp90 inhibitors may suffer from some disadvantages, including low solubility and restricted activity. To overcome these limitations, the usage of synthesized Hsp90 inhibitors has garnered much attention; for instance, purine-scaffold-based compounds were reported as effective Hsp90 inhibitors with good solubility and an acceptable level of cell permeability [67]. Purine is an aromatic heterocycle compound consisting of two fused rings. Purine derivatives have exhibited significant pharmaceutical activities such as anticancer, anti-HIV-1, and antimicrobial properties [68,69,70,71,72]. Purine and pyrimidine-based entities are essential natural heterocyclic compounds that have a critical role in several metabolic and cellular conversion processes in deoxyguanosine monophosphate (DGMP) nucleotide (AMP). Moreover, the chemical structure of several biologically important molecules, such as ATP, GTP, cyclic AMP, NADH, and 3′-phosphoadenosine-5′-phosphosulfate (PAPS) contain fused purine ring. In chemical terms, purine is a nitrogen-rich heterocyclic compound comprising two rings of pyrimidine and imidazole, and because of the lack of natural sources, they ought to be prepared through synthetic organic reactions [73,74,75].

**Figure 2:**
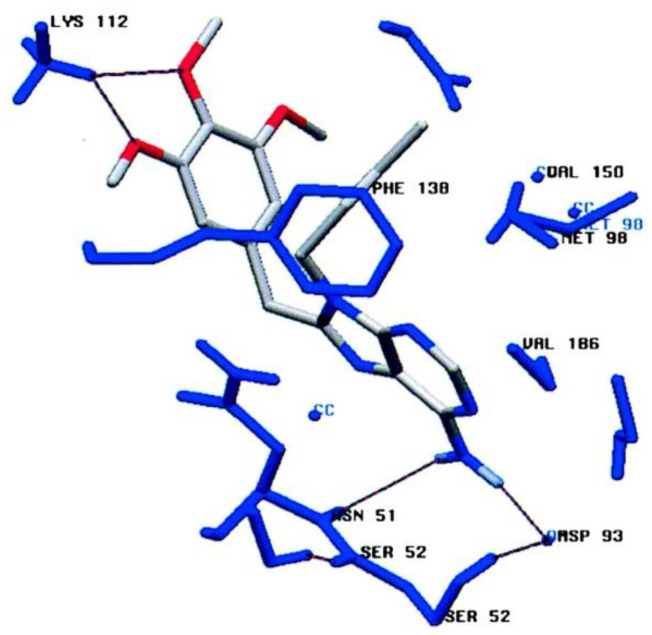
Molecular docking analysis of PU_3_ in interaction with Hsp90.

**Scheme 5:**
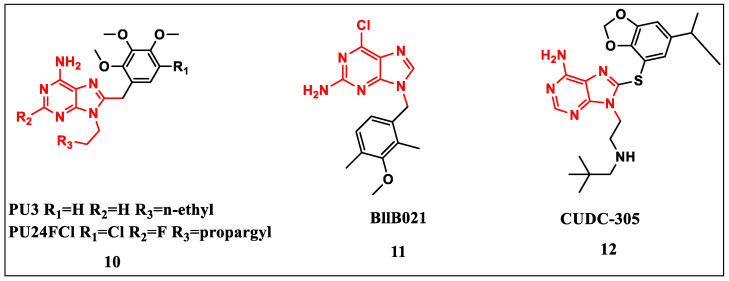
Chemical structures of PU3, PU24FCl, BIIB021, and CUDC-305.

The activity of a purine-based molecule as a synthesized Hsp90 inhibitor (PU_3_) has been investigated by assessing its interaction in Hsp90 K ADP/ADP binding site. As a theoretical outcome, this molecule covered all the necessary interactions with hosting protein; the Lys112 interacted with methoxy groups, and the hydrophobic pocket was occupied (Figure 2) [76]. The purine derivatives PU_3_ and Pu24FCl (Scheme 5) have been introduced as potential small-molecule Hsp90 inhibitors through binding to the Hsp90 ADP/ATP site. The trimethoxy phenyl functional group of Pu24FCl makes it suitable to bind into the phosphate region of the host protein. Bao et al. reported a purine-based Hsp90 inhibitor with potential oral activity, named CUDC-305 (**12**), it is a unique compound due to its: ability to extremely privilege into the tissue of the brain, prolonged duration in intracranial tumors in animal models, and function in intracranial glioblastoma models; these attractive features may be raised from its fascinating high lipophilicity (clog P of 4.0) (Scheme 5) [77].

A family of 8-arylsulfonyl analogs of PU_3_ (**10**) have been synthesized to evaluate the influence of the aryl part on inhibitory function and are being introduced as a purine-based Hsp90 inhibitor with selective activities which successfully enters clinical trials [78,79,80,81]. BIIB021 (**11**) has been the first fully synthetic Hsp90 inhibitor that moved in clinical trials endowed with its special properties that facilitated its formulation and bioavailability improvement; it binds to Hsp90 with high affinity and inhibits tumor growth. In phase I clinical trials, BIIB021 exhibited well-tolerated and good antitumor activity [82]. Therefore, this most developed purine-based compound, entered phase II clinical trials for GIST treatment. Pharmacokinetic parameters for BIIB021 600 mg, the mean C_max_ was 1.5 µmol and the mean AUC was 2.9 µmol h; a C_max_ > 1.5 µmol being associated with a decrease in standardized uptake value (SUV_max_) [83].

#### 2.2.2. Coumarin-Based Structures

**Scheme 6:**
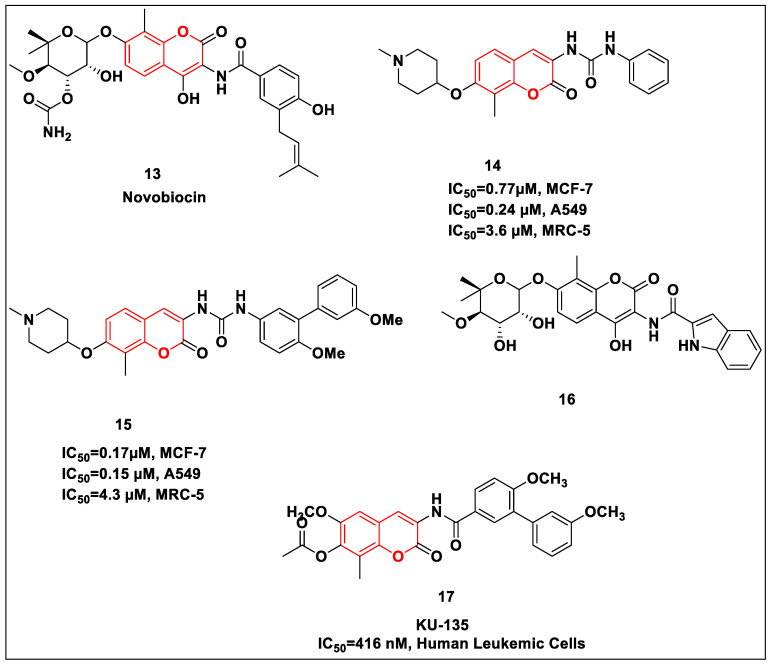
Hsp90 inhibitors containing coumarin motif.

Coumarin and its derivatives are important heterocyclic molecules endowed with various biological activities, such as platelet aggregation inhibition, antibacterial effects, and anticancer activity [84,85,86,87,88]. Novobiocin (**13**) is one of the first established organic compounds with a coumarin core that functioned as an Hsp90 inhibitor; it is a natural product with significant antibacterial DNA gyrase activity. However, its low efficiency in degrading Hsp90 clients (IC_50_ = ~700 µM) has discontinued more evaluations; therefore, research has mainly focused on structure-activity relationship (SAR) studies to identify other coumarin-based compounds with stronger inhibitor activity [89]. Modification of the 3-position of coumarin, from amide in novobiocin to urea, creates a new link to the hosting protein. The chemical structure of modified coumarin (**14**) is depicted in Scheme 6. 

Among coumarin derivatives, compound **15** (Scheme 6) did not show any Hsp90 inhibitory activities, but additional investigations revealed its ability to interrupt MAPK signaling pathway by inhibiting the level of p-ERK and p-MEK; this function would be useful in anticancer activities [90]. Blagg et al. illustrated that the 4-hydroxyl and the 3′-carbamate functional groups of novobiocin have proven to be detrimental as their essential components for Hsp90 inhibitory activity by SAR studies. Therefore, indole moiety replacement in compound **16** significantly increased its activity (Scheme 6), more than 500 times that of novobiocin [91]. Furthermore, Shelton et al. synthesized KU135 as an Hsp90 inhibitor agent with anti-proliferative activity. The results showed that this compound can degrade Hsp90 client proteins through signaling pathways, a process that has stronger anti-proliferative effects than the N-terminal Hsp90 inhibitor 17-AAG. Notably, the explorations on this Hsp90 inhibitor demonstrated that this compound could inhibit G2/M cell cycle and have mitochondria-mediated apoptosis effects [92].

**Scheme 7:**
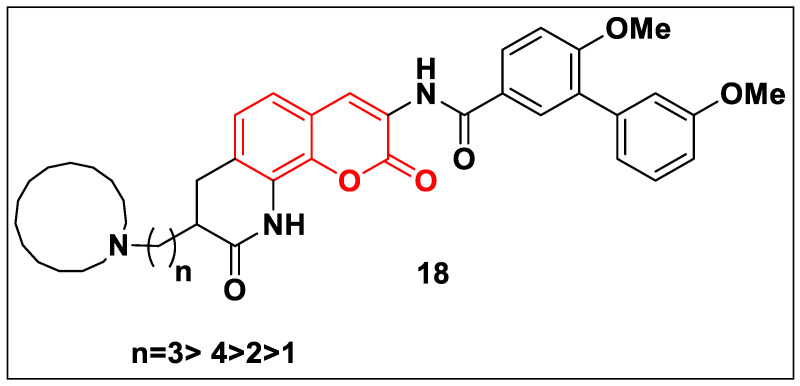
Chemical structure of ring-bound novobiocin analogs synthesized and evaluated by Garg et al. [93].

Garg et al, produced different analogs of ring-bound novobiocin (Scheme 7) wherein SAR and computational studies illustrated that when lactam was in α position, it produced more effective analogs than sugars. Activity of these derivatives was assessed as anti-proliferative agents against SKBr3 and MCF-7 cell lines. Among these derivatives, the cyclohexylamine analog demonstrated the best inhibitory effect with IC_50_ compared to bicycloalkyl and tricyclic amino analogs (0.35 μM against MCF-7 and 0.2 μM against SKBr3 cells) [93].

**Scheme 8:**
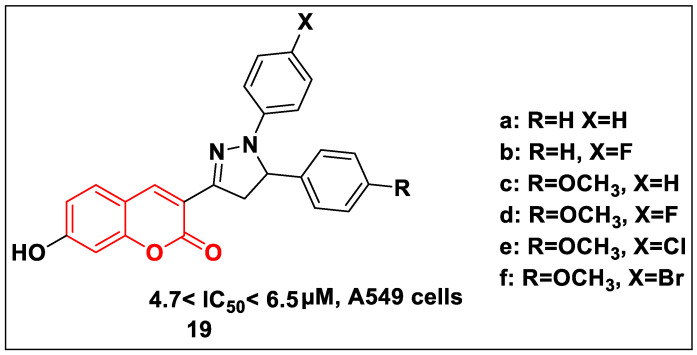
Chemical structures of pyrazoline containing coumarin compounds, reported by Wei et al. [94].

Wei et al. reported some assorted coumarin compounds comprising the pyrazoline functional group, (compound **19** (a–f), Scheme 8) and evaluated their anticancer activity through several biological assays. Based on docking study results, all of these compounds are located in the active site of the N-terminus of Hsp90. Among these six derivatives, the structure of 19a exhibited higher binding energy and Hsp90 inhibitory function (IC_50_ = ~4.7 µM). All these derivatives reduced the viability of A549 lung cancerous cells, without any necrosis induction on them as they stimulated the apoptosis with blocking effects on the autophagic flux of HCP1 in A549 lung cancerous cells [94].

#### 2.2.3. Quinolone-Based Structures

**Scheme 9:**
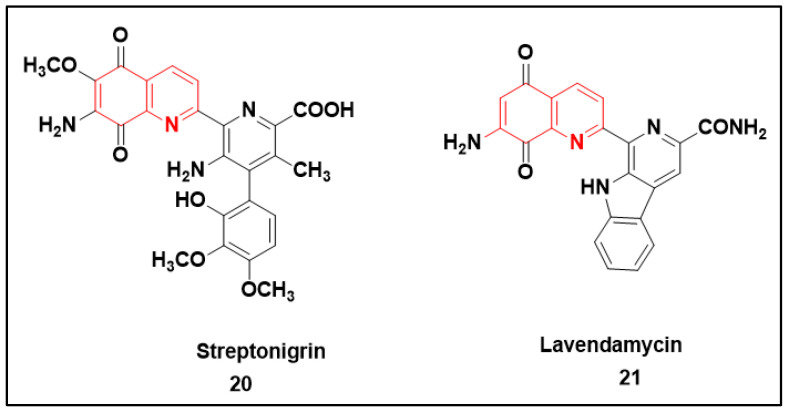
Chemical structures of quinolones-based HSP90 inhibitors: Streptonigrin and Lavendamycin.

Quinolines are one of the most important nitrogenous heterocyclic compounds that have been extensively examined due to their widespread pharmacology appliances, such as anti-malarial, antitumor, anti-parasitic, antibacterial, anti-asthma, antidiabetic, anti-inflammatory, antiplatelet, and antihypertensive activities [95,96,97,98,99,100,101,102]. Streptonigrin (isolated from *Streptomyces flocculus*) and lavendamycin have already been known as antimicrobial and antitumor compounds with quinoline skeleton (Scheme 9); they create efficient interactions with targets to act as a cancer chemotherapy agent and as Hsp90 inhibitors [103,104,105].

**Scheme 10:**
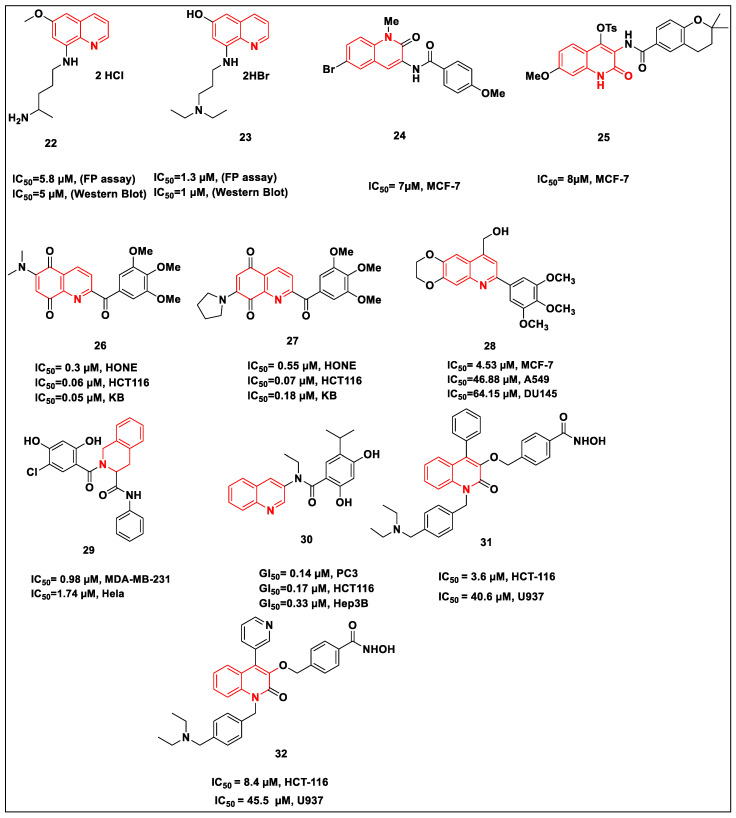
Hsp90 inhibitors containing quinoline motif.

The chemical structures of synthesized quinolones-core organic compounds with Hsp90 inhibitory potentials properties have been investigated; their chemical structure is depicted in Scheme 10, named 22 to 32. Ganesh and co-workers reported the modest Hsp90 inhibitor activity of compound **22** (Scheme 10). Several quinoline-based organic compounds were synthesized and their activities were evaluated in micromolar concentrations using cell-based Western blot (WB) and fluorescent polarization (FP) techniques [106].

Studies of SAR, optimization of structures, and re-synthesis of several aminoquinoline compounds indicated their low Hsp90 inhibitor activity. However, the synthesized compound **23** (Scheme 10) exhibited high activity, with IC_50_ of ~0.73 µM and 1 µM in the low micro-molar range obtained in FP and WB assays, respectively. These compounds, with their simple chemical structures and facile synthesis pathways, are recognized as a series of new Hsp90 inhibitors [106]. Audisio and co-workers synthesized a novel array of 3-(*N*-substituted) aminoquinolin-2(1H)-one derivative and evaluated their anticancer activity using cell proliferation and flow cytometry, including biological assays.

Among these synthetic derivatives, compounds **24** and **25** (Scheme 10) offered the most effective inhibitory activity against various genes, such as Raf-1, HER2, CDK4, and estrogen receptors. It was indicated that compound **24** could stimulate apoptosis in MCF-7 breast cancer cells by activating caspases and subsequent division of poly (ADP-ribose) polymerase (PARP) and inhibiting the growth of all tumor cell-independent cell lines with growth inhibition of 50% (GI_50_) values in the range of 2 to 32 μM. The examined cell lines included MCF-7, T47D, IRGOV-1, Ishikawa, HT-29, Caco-2, and MDA-MB-231. In addition to these properties, only compound **24** was identified as mediated cell death inductor in a p23-independent procedure; the p23 was a small and important co-chaperone for the Hsp90 chaperoning pathway [107]. 

A series of 2-aroylquinoline-5,8-diones have been synthesized and their potential biological activities have been evaluated by Nepali and coworkers [108]. Among these Hsp90 inhibitors, compounds **26** and **27** could inhibit the growth of cancerous cells (IC_50_ = ~0.14 (compound **26**) and 0.27 μM (compound **27**). Moreover, compound **27** displayed an IC_50_ of ~5.9 μM to inhibit tubulin polymerization as it persuaded the degradation of Hsp70 and Akt protein through WB analysis. Different quinoline analogs have been reported as Hsp90 inhibitors with a cytotoxic function against cancerous cell lines. Accordingly, cytotoxic derivatives encompassing alcohol functional groups exhibited significant activity against MCF-7 cells. Among these evaluated compounds with anti-proliferative activity, compound **28** distinguished itself as the most effective analog in the degradation of Her2 protein, a client protein of Hsp90. The possible state of interaction between compound **28** and the N-terminal ATP binding pocket of Hsp90 was demonstrated by molecular modeling studies [109]. 

Liang et al. synthesized two new series of compounds of *N*-(5-chloro-2,4-dihydroxybenzoyl)-1,2,3,4-tetrahydroisoquinoline-3-carboxamides as Hsp90 inhibitors, which illustrated acceptable anti-proliferative activities against MDA-MB-231 and HeLa cell lines. Compound **29** unveiled strong cytotoxicity with inhibitory effects against the molecular proliferation of MDA-MB-231 (IC_50_ = ~0.98 µM) and HeLa (IC_50_ = ~1.74 µM). Moreover, the effective intracellular interaction of compound **29** with Hsp90α in 293T cells was confirmed through isothermal dose-response fingerprint curves. The induction activity of compound **29** in the degradation of CDK4, Her2, Cdc-2, and C-RAF Hsp90 client proteins was assessed by WB evaluation on MDA-MB-231 breast cancer cell lines [110].

**Figure 3:**
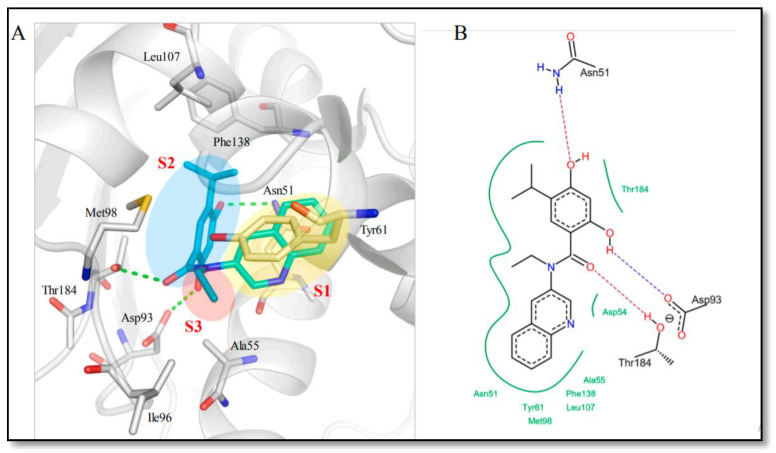
MD analysis of compound **30** in interaction with Hsp90. (**A**) compound **30** (blue) is anchored within the Hsp90 (gray) binding site. The three distinct sections of MPTG0G256 that are located in the S1, S2, and S3 sites, are colored as yellow, blue, and red, respectively. Interacting residues are shown as sticks and labeled as indicated. Hydrogen bonds are indicated by dotted green lines. (**B**) 2D representation of compound **30** docked in Hsp90. Green lines show regions of hydrophobic interactions. Interacting residues are labeled as indicated. Adapted from Ref. [111] with permission. Copyright 2019 Elsevier.

Molecular docking and dynamic (MD) analyses on the complex of compound **29** and Hsp90 displayed its effective binding to Hsp90 through the interaction of its benzyl amino moiety with the residue Phe138, leading to the formation of a Π-stacking interaction [110]. Nepali et al. reported different fused quinoline-resorcinol compounds with powerful inhibitory activity against Hsp90. Through MD analysis of synthesized compounds in interactions with the Hsp90 chaperone protein receptor (Figure 3), compound **30** was determined as the ideal candidate. It interacted with the amino acid residues of the Hsp90 chaperone protein, resorcinol ring of compound **30**, 1,3-dihydroxybenzene, link to chaperone function through amide bond formation with 1,3-dihydroxybenzene. In vitro studies indicated the effective cell growth inhibitory effect of compound **30**, as one of the most active entities through in silico studies, against HCT-116 (colon), Hep3B (liver), and PC-3 (bone metastasis) cell lines [111].

Relitti and co-workers synthesized various quinolone-based organic compounds as histone deacetylase 6 (HDAC6), wherein compounds **31** and **32** were introduced as the most promising active molecules against HDAC6. In addition, they displayed potent activity in cellular studies with development of inhibition against human cancer cell lines, HCT-116, and histiocytic lymphoma (U9347). It was an effective compound against tumor cells via apoptosis induction [112]. 

#### 2.2.4. Triazine and Triazolothione-Based Structures

**Scheme 11:**
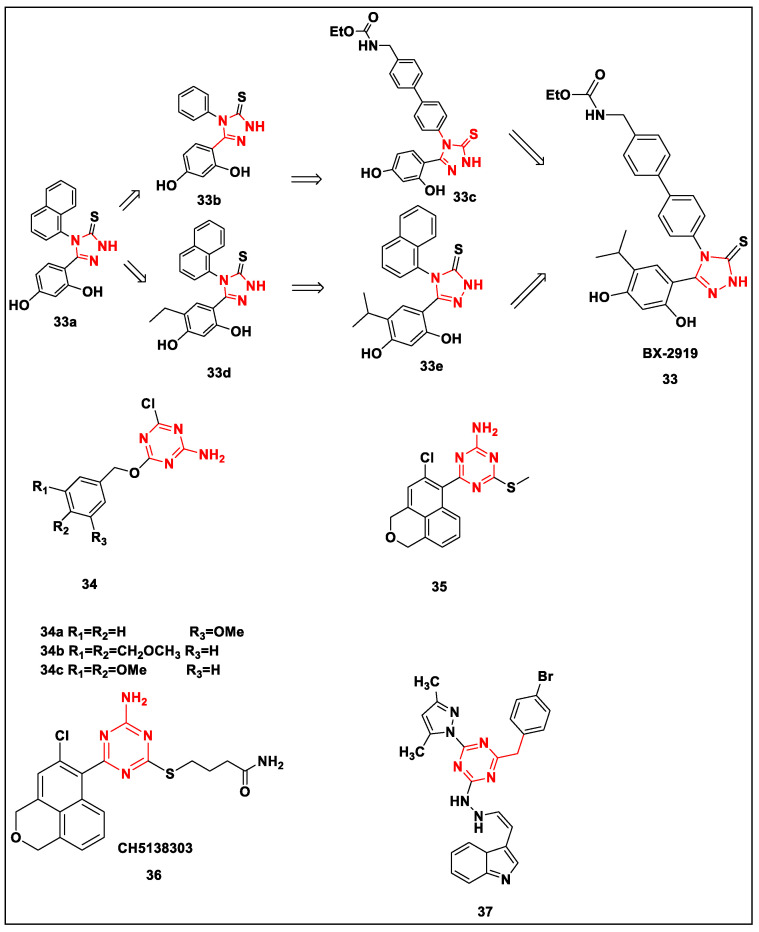
Hsp90 inhibitors containing triazine and triazolothione motif.

Triazine is an organic heterocyclic compound with a 6-membered ring containing three carbon atoms and three adjacent nitrogen atoms. Pharmacology studies have revealed that triazine derivatives have significant activity as antimicrobial, antituberculosis, anticancer, antiviral, and antimalarial agent [113,114,115,116,117]. BX-2919 was discovered by Feldman et al. as an Hsp90 inhibitor during HTS, through a high-throughput screening assay that was performed to identify Hsp90 inhibitors that compete with GA binding. The resorcinol **33a** analog (Scheme 11) was identified as one of Hsp90 inhibitors optimized by applying parallel paths on its aryl rings. After assessing the strength of a large number of aryl compounds, ethyl carbamate 33c was found as one of the best candidates. Among the resorcinol ring alternatives, the ethyl analog (**33d**) increased relative strength; finally, the isopropyl analog (**33e**) was the optimal alternative in this situation. A combination of optimal alternatives with the BX-2819 was examined. This compound could bind strongly to Hsp90 (IC_50_ = ~41 nM) to inhibit GM-Bodipy binding, which was less than both above-mentioned compounds 17AAG (IC_50_ = ~350 nM) or radicicol (IC_50_ = ~87 nM) [118].

Seo et al. reported a set of 2-amino-1,3,5 triazines containing a tricyclic part as Hsp90 inhibitors with high activity against gefitinib-resistant H1975 cells (Scheme 11) [119]. Several derivatives of compound **34** (Scheme 11) were synthesized, most of which entailed cell multiplication in a highly dose-dependent behavior with regularity effects on cell proliferation. Among them, compound **34a** demonstrated the highest activity as an inhibitor of cell proliferation and the product containing 2,6 dimethyl phenyl was able to reach the hydrophobic part of Hsp90 by van der Waals interactions, which led to anti-proliferative activity against H1975. Miura et al. synthesized various tricyclic molecules, including 2-amino-1 and 3,5-triazines as Hsp90 inhibitors with suitable anti-proliferative function against HCT-116 (IC_50_ = ~0.46 mM). Among these synthesized compounds, hybrid CH5015765 (35, Scheme 11) significantly improved the binding affinity (Kd = 3.4 nm) [120].

The structural properties are involved in the possible interactions with Hsp90 at the active region, thus the fabricated tricyclic molecules should be precisely analyzed. Consequently, it was indicated that the hydrogen bonds can be formed between the amino group on the two positions of the triazine core and the carboxylic group of Asp93. A hydrophobic interaction can be formed between the methyl thio group at the 4-position and Ile96/Met98. Additionally, a hydrophobic portion can interact with the side chains of Leu107, Phe138, and Val150. Finally, the ether oxygen may bind to the remnant Phe138 and Asn51 [121]. Compound **36** (CH5138303) showed potent inhibitory activity against HCT-116, in vitro. Its efficacy and chemical properties led to reducing the phosphorylation and protein level of several Hsp90 client proteins. By further improving the chemical structure of tricyclic molecules, a category of triazines derivatives could be produced via the functionalization of the sulfur atom in compound **36** with hydrogen bond acceptor functional groups. They are primed to interact with the Lys58, which concerned in several hydrogen bonds with geldanamycin as a natural and specific inhibitor of Hsp90. In general, the derivative of compound **36** exhibited significant antiproliferative activity, in vitro and in vivo (Kd = 0.48 nM) [122].

The antitumor activity of compound **37** as an Hsp90 inhibitor agent was reported by Zhao et al. [123]. Their interaction with Hsp90 was studied by surface plasmon resonance (SPR) assay; consequently, the results indicated the binding of compound **37** into the N-terminal of the protein with a very extraordinary binding mode with respect to the old inhibitors of Hsp90. Its anti-proliferative function was evaluated in vitro and in vivo. Accordingly, the results obtained from in vitro assessments demonstrated that these compounds could inhibit the molecular proliferation of BT-474 (IC_50_ = ~8.9 mM), SK-BR-3 (IC_50_ = ~7.1 mM), A549 (IC_50_ = ~7.5 mM), K562 (IC_50_ = ~8.6 mM), and HCT-116 (IC_50_ = ~6.7 mM). After in vivo assessments, it was revealed that these compounds could obstruct the increase in proliferation of tumors that increased with a dose-dependent behavior in the BT-474 [123].

#### 2.2.5. Isoxazole-Based Structures

One of the prominent heterocyclic organic compounds comprises the isoxazole ring, a 5-membered ring, with adjacent oxygen and nitrogen atoms. Various chemical structures/compounds encompassing the isoxazole moiety have shown attractive biomedical and pharmaceutical potentials, especially for cancer therapy [124,125,126,127,128,129,130]. Medicinal chemistry studies have revealed that the inclusion of isoxazole in the chemical structure of Hsp90 inhibitors could lead to improved efficacy, decreased toxicity, and enhanced pharmacokinetics profiles. Aromatase is an enzyme for the conversion of androgenic hormone into estrogen; thus, considering the high-level expression of aromatase in breast tissue, intense generation of estrogen can cause breast cancer. Isoxazole derivatives could stop the conversion of androgen into estrogen by inhibiting the aromatase enzyme, thus serving as anticancer drugs [131].

Apoptosis is an extremely regulated process enabling cells to destroy themselves and kill unwanted cells; this has a crucial role in homeostasis development. Isoxazoles as an inducer of apoptosis causes the elimination of or discontinues the progression of cancer [132]. Enzymes of protein tyrosine phosphatases are involved in mechanisms of cellular signaling by regulating the levels of phosphorylation in a clear tyrosine residue in proteins or peptides, controlling various functions of cells, including metabolism, migration, survival, adhesion, and multiplication. Mutations in proteins can cause cancers. In this context, isoxazole derivatives can attach to the ATP-binding region of the protein kinase and hinder the signaling cascade, resulting in cell cycle arrest and apoptosis [133].

Many preclinical studies have indicated that Hsp90 inhibition is associated with anti-neoplastic effects; these properties motivated researchers to find novel small-molecule containing isoxazoles as Hsp90 inhibitors. Their capability to bind with the NH_2_-terminal nucleotide-binding region of human Hsp90 was found extremely strong along with inhibitory effects on the multiplication of growth cells through inducing the arrest of G1-G2 and apoptosis. Pharmacological compounds containing isoxazole are active against different examined cell lines of tumors, especially breast cancer, which stimulated numerous efforts to design and synthesize innovative isoxazole derivatives [134].

**Scheme 12:**
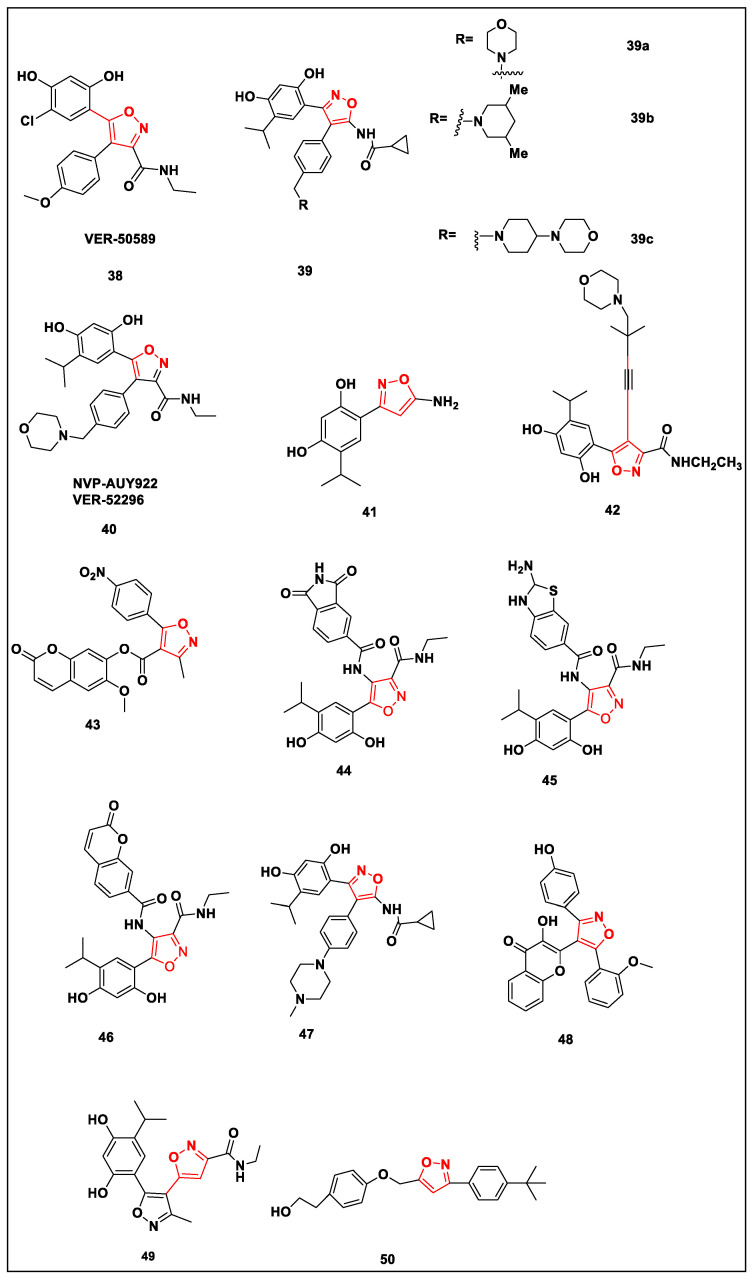
Hsp90 inhibitors containing isoxazole motif.

Eccles et al. synthesized organic compounds containing isoxazole, such as VER-50589 (Scheme 12). Their Hsp90 inhibitory activity was assessed through in vivo studies, wherein these small synthetic molecules exhibited strong inhibitory activity. The results were found comparable with the activity of the clinically approved drug 17-AAG, an analog of geldanamycin; additionally, their lipophilicity renders them capable of easily penetrating the cell membrane. The encouraging outcomes illustrated the value of a structure-based design and optimization process on the way to achieve a potential clinical candidate. Prodigious findings motivated more research on the optimization of their potency, pharmacokinetic, and pharmacodynamic properties via structural modifications, which led to the discovery of VER52296 (NVPAUY922) (Scheme 12). The anticancer activity of VER52296 was investigated against cell lines, namely, HCT-116, Hun7, and SW620, by applying 3-(4,5-dimethylthiazol-2-yl)-2,5-diphenyltetrazolium bromide tetrazolium (MTT) assay. The obtained results demonstrated high cytotoxic activity with IC_50_ values lower than 20 μM (IC_50_ = ~21 nM and Kd = 1.7 nM), besides the low glucuronidation metabolite levels. Thus, VER52296 was introduced as a potential anticancer drug, and it is under evaluation in a second phase clinical trial [135].

A set of Hsp90 inhibitors comprising *N*-(isoxazol-5-yl) amides structures presented unique pharmacokinetic characteristics as well as high antitumor activities (both in vitro and in vivo). Compounds **39a**, **39b**, and **39c** exhibited the GI_50_ lower than 0.1 µM; thus, studies have been performed on their activity against the various cell lines. They displayed potential cytotoxicity with common GI_50_ which indicated their extensive antitumor activities [135]. Chen et al. reported various small molecules comprising isoxazole rings and identified them by X-ray crystallographic techniques. Their Hsp90 inhibitory effects were evaluated, and compound **40** exhibited potent inhibitory effects on Hsp90 at cellular/molecular levels; moreover, in vivo assessments showed its remarkable capability for inhibiting the growth of tumors. The ratio between treated and control (T/C) value of compound **41** was equal to 18.35% at 50 mg/kg, which was found to be nearly twice as strong as NVP-AUY 922 as a traditional Hsp90 (T/C values at 50 mg/kg reach 34.06%) [136]. 

Sun et al. reported a set of isoxazole scaffolds associated with alkynes to study their inhibitory activity on the human Hsp90 protein as well as their anti-proliferative function against the examined cell lines of tumors. During conservation, it was found that the substitution of alkynes at C-4, in compound **42**, could induce nice cation–π bond interaction with the Lys58 residue of the Hsp90 protein, along with the interaction of the resorcinol hydroxyl and C-3 amide groups. Based on the results, a set of 4-alkynylisoxazole structures were synthesized with 3,5-substitutes and their binding affinity to Hsp90 protein was also indicated. In addition, their anti-proliferative effects were evaluated against five cancerous cell lines, such as A549, human basal alveolar epithelial cell adenocarcinoma, K562, human immortal myeloma leukemia line, MCF-7, and the cell line of most breast cancers. Promising results were obtained, wherein compound **42** demonstrated an IC_50_ value of ~0.066 µM [137].

Shi et al. synthesized a set of scopoletin-isoxazole and scopoletin-pyrazole hybrids and their anticancer effects were evaluated against SW620, Hun7, and HCT-116. Scopoletin-isoxazole derivatives illustrated higher cytotoxic activity in comparison with scopoletin-pyrazole hybrids (IC_50_ ≤ 20 μM). Impressively, compound **43** showed anti-proliferative effects similar to sunitinib, the FAD-approved drug, with IC_50_ = ~8.76–9.83 μM against SW620, Hun7, and HCT-116, and it disclosed low toxicity on normal HFL-1 cells (IC_50_ = ~90.9 μM) [138]. Sadeghi et al. studied the potential Hsp90 inhibitor activity of 50 organic molecules, including 3,4-isoxazolediamides functional groups; they discovered that three compounds, **44**, **45**, and **46**, could be considered as promising target molecules for further evaluations [139]. They also introduced compound **47** as an efficient Hsp90 inhibitor after the assessment of several isoxazoles-based compounds [140]. Additionally, several chromone-linked isoxazole compounds were synthesized, and their ER-α and their anti-proliferative activity against MCF-7 were evaluated. The replacement of isoxazole’s positions of 3 and 5 with phenyl ring and 3-hydroxychromone increased their anticancer activity. Compound **48** displayed cytotoxicity against ERα-positive (MCF-7) with IC_50_ of 32 µg mL^−1^ by linking with a key residue of Glu353 and Arg394. These residues were stabilized by forming H-bonds of 4-hydroxyl phenyl in 4HT (4-hydroxytamoxifen) through molecular docking studies investigations [141].

Jung et al. reported a variety of isoxazole-containing compounds as Hsp90 inhibitors. Their capability of client protein degradation as well as ATPase performance was analyzed through in vitro cytotoxic assessments against cancerous cell lines. The targeted compounds were assessed for their possible antitumor effects in tumor xenograft models in vivo. Molecular modeling studies demonstrated the binding mode inside the inhibitors and N-terminal ATP binding pocket. To evaluate the antitumor effects of isoxazole-containing compounds, their cytotoxicity was analyzed against A2780 (ovarian cancer) and HCT-116 (colon cancer). Consequently, the IC_50_ of compound **49** for ATPase, Her2, A2780, and HCT-116 were found to be 0.284, 0.028, 0.043, and 0.014 µg, respectively [142]. Aissa and co-workers reported the synthesis of 3, 5-disubstituted isoxazoles and 1,4-disubstituted triazoles and evaluated their possible anticancer/hemolytic activities. The anticancer derivatives of isoxazole exhibited an active apoptotic trend in the glioma U251-MG and T98G. These derivatives illustrated significant anti-proliferative function against most human glioblastoma cancerous cell lines (U87) in a dose-dependent manner wherein compound **50** (IC_50_ 15.2 ± 1.0 μg mL^−1^) exhibited stronger anticancer activity. Notably, the hemolytic activity of compound **53** caused hemolysis up to 40% at a concentration of 400 μg mL^−1^. These results revealed that isoxazole derivatives did not damage red blood cells and release hemoglobin, which strengthened the potential of these compounds as innovatively synthesized anticancer prototypes with low toxicity [143].

#### 2.2.6. Pyrazole-Based Structure

**Scheme 13:**
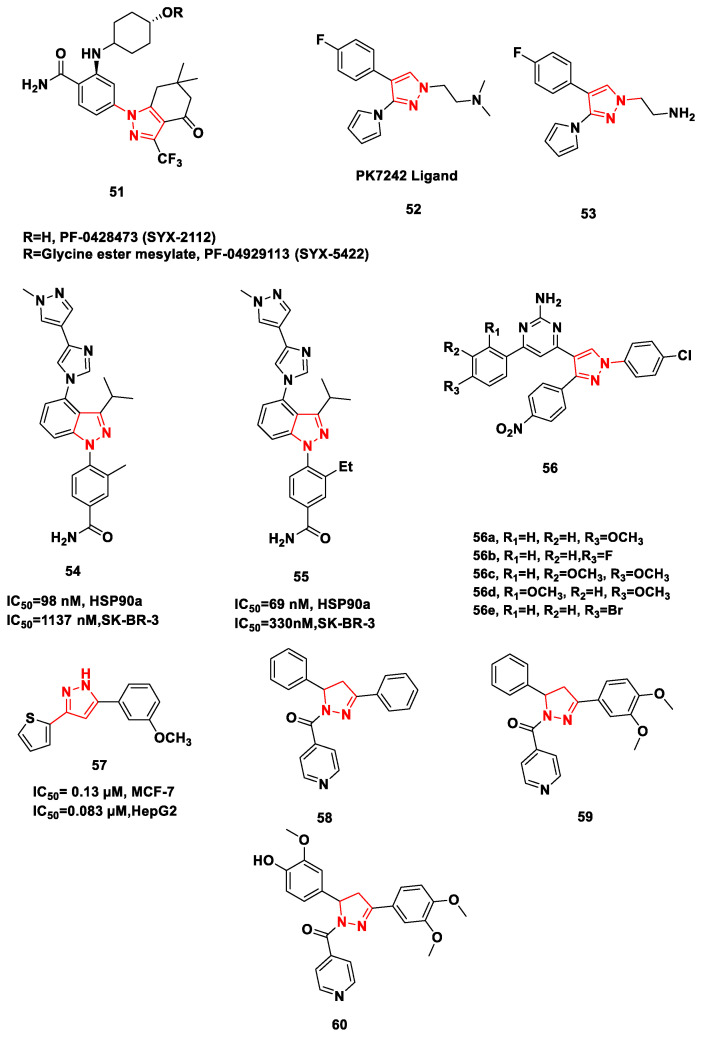
Hsp90 inhibitors containing pyrazole motif.

Pyrazole is a 5-membered ring heterocycle compound consisting of three carbon atoms and two adjacent nitrogen atoms. Pyrazole derivatives have significant activities, such as high efficacy as partial agonists in G protein-coupled and cannabinoid receptors [144,145,146,147]. Synthetic small pyrazole-fused heterocycle molecules, PF-04929113 and PF-04929113, were developed as inhibitors of Hsp90, but their restrictions/disadvantages, such as poor solubility and cytotoxicity prompted a search for the structural improvement of pyrazole-based compounds (Scheme 13) [148]. Bai et al. synthesized various fluorescent pyrazoline coumarin derivatives and investigated their anticancer activity against lung cancer cell lines in vitro. As a result, compound **52** had the strongest growth inhibition (IC50 = ~7.9 mM), with a strong fluorescence strip; subsequently, it was introduced as a potential and promising fluorescent Hsp90 inhibitor [149].

Sadeghi et al. predicted dual agents as mutant P53 activators and Hsp90 inhibitors by using docking and MD analysis; according to their published results, compound **53** was proposed as a dual-mode agent, P53 activator, and Hsp90 inhibitor [150]. Uno et al. reported various pyrazolo [3,4-b]pyridine derivatives as potential Hsp90α and Hsp90β inhibitory agents with oral availability properties. Compound **55** exhibited the same binding state of Hsp90 as an analog of pyrazole compound **54** via X-ray crystallography. Oral administration of compound **54** demonstrated potent antitumor effects on human lung cancer (NCI-H1975) in a xenograft mouse model [151]. Mettu et al. innovatively synthesized a variety of pyrazolyl 2-aminopyrimidine derivatives and evaluated their Hsp90 inhibitory and anticancer activity. Among them, compound **56e** established the highest binding affinity to Hsp90 (20 nM) with anti-proliferative function against MCF7 (IC50 = ~2.4 µM), MDA-MB-231 (IC50 = ~0.8 µM), and HCT-116 (IC50 = ~4.8 µM), in vitro. According to Western blotting (WB) analysis, two compounds (**56b**, **56e**) generated dose-dependent degradation of two client proteins (pHER2 and pERK1/2) [152]. Molecular docking studies demonstrated that the compounds **56b**–**56e** were significant Hsp90 inhibitors. These studies revealed that the para substitution on pyrazole rings A and B, especially with the p-nitro group on ring B and 2 amino groups on the pyrimidine ring, had unique consequences on the development of new Hsp90 inhibitors. The binding potential of the disubstituted pyrazolyl pyrimidine scaffold and pocket of Hsp90 had effective interactions with Thr184, Asn51, and AsH93 (a protonated form of Asp93), Asp54, and Lys58 [152]. According to the results reported by Mettu et al. compound **56e** was the most active compound; the apoptosis potential of this compound was recognized by the Annexin V assay, where it could induce mitochondrial stress that increases membrane permeability, causing induction of apoptosis in MCF-7 cells. This was demonstrated by increasing the generation of J-monomer by applying JC-1 stain. Furthermore, a change in mitochondrial membrane potential could inevitably give rise to ROS strains as evidenced by DCFDA staining. In addition, this compound could stop the subG1 phase cell cycle. Thus, compound **56e** could serve as a potential compound with anticancer activity [153].

Mohamady et al. synthesized and evaluated possible biological activities of 3,5-diarylpyrazoles derivatives against HepG2 and MCF-7 cancerous cells. Among them, compound **57** exhibited the highest cytotoxic activity on HepG2 (IC_50_ = ~0.083 µM) and MCF7 (IC_50_ = ~0.13 µM) cells, with efficient anti-proliferation effectiveness. Compound **57** induced the inhibition of tumor cell proliferation by stimulating G2 phase inhibition, obstructing client proteins, such as c-Raf, EGFR, Akt, and c-Met and increasing the levels of Hsp70 [154]. Kadasi et al. reported the preparation of a novel series of *N*-pyridoyl-2-pyrazolines and evaluated their Hsp90 inhibitory activities for possible anticancer effects. The docking simulations revealed the binding potential of the synthesized compounds in the N-terminal ATP in Hsp90; moreover, the optimized compounds had significant interactions with Asp93 and Thr184. Consequently, compounds **58**, **59**, and **60** demonstrated an effective cytotoxic activity against MDA-MB-468 with IC_50_ of 1.60, 2.8, and 12 μM, respectively. Compound **58** exhibited efficient inhibitory effect as well (IC_50_ = ~7.7 μM) against A375 as human melanoma cells [155]. 

## 3. Conclusions and Future Outlook 

Several investigations have focused on the design of inhibitors with heterocyclic structures to bring down the increased level of Hsp90 proteins in cancer or tumor cells, thus providing promising treatment strategies for cancer therapy with higher targeting and efficiency/efficacy. Hsp90 proteins have crucial roles in regulatory activity: folding, maintenance, function, and stability of various vital proteins, especially in client proteins. The inhibition of Hsp90s overexpressed in tumor cells can be considered an effective treatment strategy for various cancers. Numerous natural products and rationally designed synthetic Hsp90 inhibitors encompassing purine, pyrazole, triazine, quinolines, coumarin, and isoxazole structures can be viable candidates for further anticancer evaluations as deliberated. However, more systematic analyses and explorations are still needed for clinical applications and formulations of these compounds Hsp90 proteins have serious roles with regulatory activity in folding, maintenance, function, and stability of various vital proteins, especially, client proteins, which make them attractive candidates for cancer therapy targets. The inhibition of these Hsp90s overexpressed in tumor cells can be considered as an effective treatment strategy for various cancers and malignancies. 

In past years, several investigations have focused on heterocyclic structures, which are capable of inhibiting Hsp90 proteins, which are overexpressed in cancer or tumor cells, providing promising treatment strategies for cancer therapy with high targeting and efficiency. These Hsp90 inhibitors are attractive candidates due to their inhibitory effect against several cellular signaling pathways simultaneously in cancer cells or tumors. Natural compounds (**1**–**9**) were proposed as Hsp90 inhibitors, and some compounds were selected for animal and in-vivo studies. Compounds based on the purine structure (**10**–**12**) were synthesized which are claimed to bind to the Hsp90 inhibitors strength and inhibit tumor growth, these compounds were known as good candidates for clinical phase studies. Coumarin and its derivatives (**13**–**19**) have different biological activities such as platelet aggregation inhibition, antibacterial effects, and anticancer activity, these scaffolds also act as Hsp90 inhibitors. Modification of 4-hydroxyl and the 3′-carbamate functional groups of novobiocin in compound **16** led to an increase in its activity more than 500 times from novobiocin. 

Compounds aroylquinolone-based compounds were also categorized as Hsp90 inhibitors that prevent significant growth of cancerous cells. For example, compound **27** displayed an IC50 of ~5.9 μM to inhibit tubulin polymerization and degradation of Hsp70 and Akt protein through WB analysis. Compound **35**, hybrid CH5015765, containing the 2-amino-1,3,5-triazines was recognized as an Hsp90 inhibitor with good anti-proliferative activity against HCT-116 and excellent binding affinity (Kd = 3.4 nm). Triazines were also known as Hsp90 inhibitors with high activity and good anti-proliferative activity against HCT-116. In general, the triazine derivatives showed significant anti-proliferative activity in vitro and in vivo. Medicinal chemistry studies showed that the inclusion of isoxazole in the chemical structure of Hsp90 inhibitors can lead to improved efficacy. Isoxazole derivatives (**38**–**53**) can block the signaling cascade by binding to the ATP of the kinase protein and cause cell cycle arrest and apoptosis, investigations exposed that the isoxazole derivatives did not damage red blood cells, which strengthened the potential of these compounds as synthesized anticancer prototypes with low toxicity. Pyrazole derivatives (**54**–**64**) have remarkable activities, such as high efficiency as partial agonists at G protein and cannabinoid receptors, which make these properties useful inhibitors. This combination inhibits the proliferation of tumor cells by stimulating G2 phase inhibition. However, more systematic analyses are still needed for clinical applications and formulations of these compounds.

## Data Availability

Not applicable.
